# A Potential Link between Environmental Triggers and Autoimmunity

**DOI:** 10.1155/2014/437231

**Published:** 2014-02-12

**Authors:** Aristo Vojdani

**Affiliations:** Immunosciences Lab., Inc., 822 S. Robertson Boulevard, Suite 312, Los Angeles, CA 90035, USA

## Abstract

Autoimmune diseases have registered an alarming rise worldwide in recent years. Accumulated evidence indicates that the immune system's ability to distinguish self from nonself is negatively impacted by genetic factors and environmental triggers. Genetics is certainly a factor, but since it normally takes a very long time for the human genetic pattern to change enough to register on a worldwide scale, increasingly the attention of studies has been focused on the environmental factors of a rapidly changing and evolving civilization. New technology, new industries, new inventions, new chemicals and drugs, and new foods and diets are constantly and rapidly being introduced in this fast-paced ever-changing world. Toxicants, infections, epitope spreading, dysfunctions of immune homeostasis, and dietary components can all have an impact on the body's delicate immune recognition system. Although the precise etiology and pathogenesis of many autoimmune diseases are still unknown, it would appear from the collated studies that there are common mechanisms in the immunopathogenesis of multiple autoimmune reactivities. Of particular interest is the citrullination of host proteins and their conversion to autoantigens by the aforementioned environmental triggers. The identification of these specific triggers of autoimmune reactivity is essential then for the development of new therapies for autoimmune diseases.

## 1. Introduction

**Figure 1:**
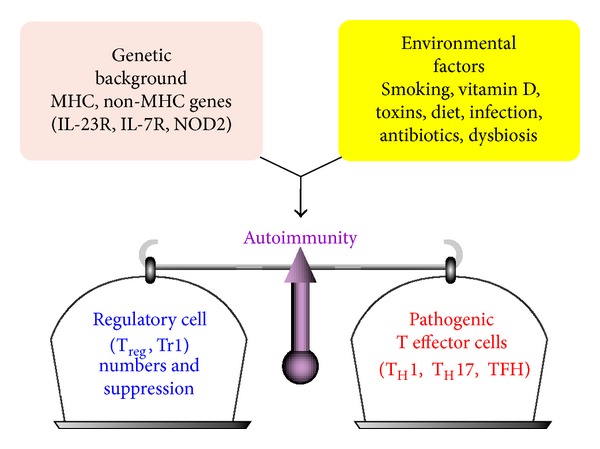
The balance of immunity. A combination of host genetic factors and exposure to environmental triggers promote the development of autoimmune disease. A balance must be maintained between the regulatory T cells and the pathogenic T effector cells.

The immune system walks a fine line to distinguish self from nonself in preserving the integrity of the host [1]. Interference with this fine line can result in overactivity to self-antigens, leading to autoimmunity. During the past 20 years a significant increase has been observed in the incidence of autoimmune disease worldwide. The etiology and pathogenesis of many autoimmune diseases remain unknown. It does appear that a close interplay between environmental triggers and genetic factors is responsible for the loss of immunological tolerance and autoimmunities [2, 3] (Figure 1). Therefore, in relation to the role of heritability in autoimmunity, genome-wide association studies reported that genetics only accounted for a minority of autoimmunity cases, and in many cases disease discordance exists in monozygotic twins [4]. For this reason, research and publications dedicated to environmental factors in autoimmunity have grown by an average of 7% every year since 1997 [2]. This includes toxic chemicals, infections, and dietary components. Indeed, detection of reactive antibodies to various citrullinated peptides and proteins in autoimmune disease is the best indication for gene-environment interactions [5].

## 2. Dysregulation of Immune Homeostasis

The full collaboration of both the innate and adaptive arms of the immune system plays a crucial role in the promotion or inhibition of autoimmune disease. Generally, to clear infections the innate immune cells can upregulate costimulatory molecules and produce a mixture of pro- and anti-inflammatory cytokines such as interleukin-1-beta (IL-1*β*), IL-12, transforming growth factor-beta (TGF-*β*), IL-23, tumor necrosis factor-alpha (TNF-*α*), and IL-6 that regulate the adaptive arm of the immune system. However, a dysregulated immune response to environmental triggers, such as pathogens, microbiota, or toxins, can initiate a chronic inflammatory response through activation of T-helper-1 (Th1), Th17, and TNF-*α* and the production of IL-17, IL-22, interferon-gamma (IFN-*γ*), and IL-21, resulting in inflammation, antibody production and tissue injury [6].

Therefore, a dysregulated adaptive immune system is at the core of the pathogenesis of autoimmune and other immune-mediated diseases. Hyperactivation of innate immune response affects the adaptive immune response as well as development effector T and B cells. Paired with defects in the regulatory T cells, this results in the breakdown of immune homeostasis and the development of autoimmunity [7].

To induce an autoimmune response in the lymph nodes, effector T cells first have to acquire a defined cytokine fingerprint and then must migrate to the appropriate target organs where they initiate tissue inflammation. The effector cells that participate in the induction of autoimmunities are IFN-*γ*-producing Th1 cells, IL-17- and IL-22-producing Th17 cells, and IL-21-producing follicular Th cells or TFH cells. It has been shown that overactivation or expansion of these newly discovered TFH cells causes antibody production and the development of lupus-like disease in an animal model [8]. In fact, high concentrations of circulating T cells that resemble TFH cells have been detected in a subgroup of patients with lupus. This increased frequency of TFH cells correlated with both disease severity and end-organ damage [9]. Unfortunately, a decrease in frequency and function of FOXP3^+^T_REG_ cell is often seen in autoimmune diseases. This decrease seems to be associated with the inflammatory environment that contributes to the dysregulation of T_REG_ cells [7].

**Figure 2:**
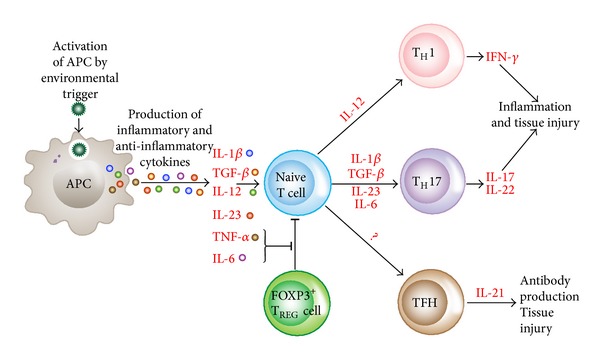
Differentiation of naïve T cells into pathogenic effector T cells. APCs can be activated by numerous factors, resulting in the release of cytokines that promote the differentiation of naïve T cells into various subsets of pathogenic effector T cells that drive inflammation, tissue injury, and autoantibody production. Segmented filamentous bacteria (SFB) can also promote the development of Th17 cells and autoimmune responses *in vivo*. Proinflammatory cytokines derived from both innate and adaptive immune cells attenuate T_REG_ cell-mediated suppression of effector T cells.

In the environment of immune homeostasis, the actions of autoreactive Th1, Th17, and TFH cells are countered by FOXP3^+^ regulatory T cells that produce TGF-*β* and IL-10. But in an inflammatory milieu the deletion of different transcription factors results in the generation of T_REG_ cells that are unable to suppress the autoreactive T cells (Figure 2).

Thus, tight control of autoreactive T cells, in particular TFH cells, by T_REG_ cells is necessary to suppress the development of autoimmune lupus-like disease.

In order to induce long-lasting remission of immune-mediated diseases, two important factors have to be in place: controlling the inflammatory environment and boosting the frequency and function of FOXP3^+^ regulatory T cells.

## 3. Toxicants and Autoimmunity

**Figure 3:**
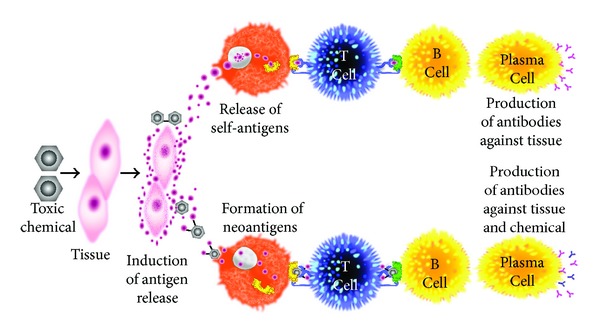
Putative mechanism of chemical-induced autoimmunity.

A number of experimental studies and clinical reports have shown that autoimmune reactivity and/or autoimmune diseases are induced in humans and chronic exposure to various chemicals in animal models. These were summarized by Bigazzi in 1997 [10]. Furthermore, very recently, this role of environmental chemicals, in particular, the induction of autoimmunities by toxicants, was summarized by Pollard et al. [11] in his paper, “Toxicology of autoimmune diseases.” The mechanism of toxicant-induced autoimmunity is described by either toxicant induction of aberrant cell death making the hidden cellular material available to anti-gen presenting cells [12, 13] or by immune reactions to xenobiotics through covalent binding of chemicals or haptens to human tissue proteins and formation of neoantigens [14] (Figure 3). This is due to the fact that reactive organic compounds most often bind covalently; that is, their electrophilic properties enable them to react with protein nucleophilic groups such as thiol, amino, and hydroxyl groups. Examples of such reactive, haptenic compounds that frequently lead to sensitization after dermal contact or inhalation are toluene diisocyanate, trimellitic anhydride, phthalic anhydride, benzoquinone, formaldehyde, ethylene oxide, dinitrochlorobenzene, picryl chloride, penicillins, and D-penicillinamine. Sensitizing metal ions react somewhat differently in that they oxidize proteins or form stable protein-metal chelate complexes by undergoing multipoint binding with several amino acid side-chains [12]. For example, in regard to nail polish and its association with primary biliary cirrhosis (PBC), halogenated compounds could bind to mitochondrial proteins, changing their immunogenicity and inducing antimitochondrial antibodies [10, 15, 16].

In contrast to haptenic compounds, most xenobiotics eliciting adverse immune reaction are unable to bind to proteins when entering the body; however, they can do so after conversion to reactive metabolites. These xenobiotics can be considered as prohaptens, which, after metabolization, manage to bind to human tissue proteins and induce antibody production against both the haptenic chemicals as well as tissue proteins.

Another mechanism is the activation of toll-like receptors by xenobiotics. This predisposes individuals to toxicant-induced inflammatory cytokine production, which exacerbates autoimmune diseases [17].

These and other mechanisms of action were explored in relation to exposure to organic solvents as a risk factor for autoimmune disease in a very extensive systemic review of literature and meta-analysis [18]. After reviewing a total of 103 articles and the inclusion of 33 in the meta-analysis, it was concluded that (1) exposure to organic solvents was associated with systemic sclerosis, primary systemic vasculitis, and multiple sclerosis (MS) and (2) individuals who carry genetic factors for autoimmunities should avoid any exposure to organic solvents in order to avoid increasing their risk for autoimmune diseases.

**Figure 4:**
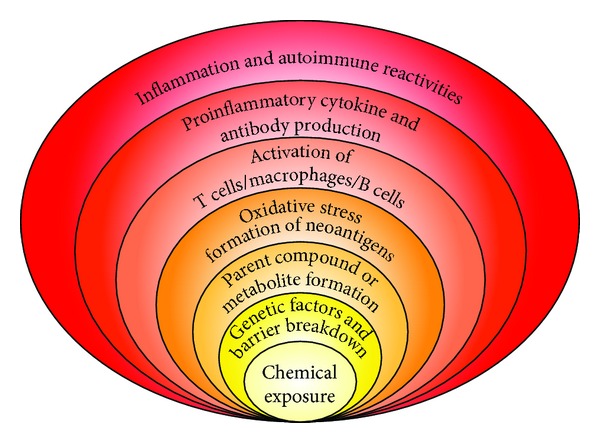
Potential molecular mechanisms implicated in chemical-induced autoimmune reactivities.

In addition to the above mechanisms of actions (as shown in Figures 3 and 4), these autoimmune responses and diseases can be induced by solvents and other environmental chemicals through a variety of effects at the biochemical and cellular levels.

- Chemicals are capable of altering cellular proliferation, Th1, Th2, Th3, Th17, apoptosis, and tissue-specific function.
- Chemicals are capable of inducing protein or lipid adducts which activate Th17 cells and induce the production of IL-17 and IL-21.
- Chemicals can activate HSP90 and induce production of anti-HSP90 autoantibodies.
- Chemicals are capable of inducing DNA-hypermethylation and change in cellular functions.
- Chemicals can increase ROS production and the induction of DNA-fragmentation.
- Chemicals may compete with thyroid hormones or interfere with iodine transportation and induce oxidative stress that leads to an inflammatory response to the thyroid gland.
- Chemicals not only stimulate the release of reactive oxygen species but also stimulate the synthesis of nitric oxide by nitric oxide synthase [18].

Finally, modification of DNA methylation is an additional mechanism by which environmental triggers induce changes in gene expression. For example, environmental pollutants, cigarette smoke and alcohol consumption have been advocated for autoimmunity incidence due to their links with the induction of DNA methylation [10, 19].

Overall, the precise mechanisms responsible for the development of environmentally induced autoimmune disorders are unknown. Additionally, mechanisms involved in the initiation of a disease process might differ from mechanisms responsible for exacerbation of the established illness. Therefore, one or more of these mechanisms either individually or jointly can have strong effects on the development of autoimmune reactivity, which may then be followed by autoimmune disease (Figure 4).

## 4. Induction of Autoimmunities by Infection

Although some infections can protect individuals from specific autoimmune diseases, infectious agents play a pivotal role in the induction of autoimmune disorders. The question of how infectious agents contribute to autoimmunity has continued to be of interest to clinical and basic researchers and immunologists in general [20].

An autoimmune disease can be induced or triggered by infectious agents, which can also determine its clinical manifestations. Most infectious agents, such as viruses, bacteria, fungi, and parasites, can induce autoimmunity via different mechanisms. In many cases, it is not a single infection but rather the “burden of infections” from childhood that is responsible for the induction of autoimmunity [20].

Almost every autoimmune disease is linked to one or more infectious agent. During the past 50 years molecular techniques have been utilized to explore the interaction between infections and autoimmunities [20–23]. One of the classical examples of this relationship is rheumatic fever, which presents several weeks after infection with beta hemolytic streptococcus. Molecular resemblance between the bacterial M5 protein and human *α*-myosin results in a breakdown of immunological tolerance and antibody production against *α*-myosin in genetically susceptible individuals [21, 24]. In the case of antiphospholipid syndrome (APS), anti-cardiolipin and anti-*β*_2_-glycoprotein I pathogenic (*β*_2_GPI) antibodies are detected. Although there is molecular mimicry between *β*_2_GPI and infections, such as cytomegalovirus, *Haemophilus influenza, Neisseria gonorrhoeae*, rubella, toxoplasma, and tetanus toxoid, and IgM antibodies against them have been detected, the direct connection between these infections and APS has not been established [24]. Another example of associating infection with autoimmune disease is type 1 diabetes. Type 1 diabetes is an autoimmune disease resulting from the destruction of *β*-islet cells by autoreactive T cells and the concomitant release of various islet cell antigens [21, 25]. The appearance of antibodies against glutamic acid decarboxylase 65 (GAD-65) and tyrosine phosphatase precedes the onset of the disease by 5–10 years [26, 27]. Several lines of evidence link infections with type 1 diabetes.

1. A search on PubMed using the keywords “association of viruses with type 1 diabetes” produces close to 1,400 manuscripts.
2. Enteroviruses such as coxsackie B4 virus and rotavirus, the most common cause of childhood gastroenteritis, not only share homology with GAD-65, but can cause the precipitation of type 1 diabetes when introduced. Higher levels of anti-coxsackievirus and rotavirus antibodies are detected in sera from patients with recent onset of type 1 diabetes [28, 29].
3. Using PCR technology, coxsackie B4 virus was detected in islet cells of 65% of patients versus only 6% of controls [30].
4. Inoculation of the virus to genetically susceptible strains of mice resulted in insulitis and diabetes, fulfilling Koch's postulates [21, 31, 32].
5. Both DNA and RNA viruses are capable of initiating antiviral responses that cross-react with insulin, GAD-65, and other islet cell antigens [21, 33, 34].

Altering the balance of gut microbiota toward either a tolerogenic or nontolerogenic state using antibiotics or probiotics may influence the development of type 1 diabetes [35, 36]. Therefore, just as with their viral counterparts, there is sufficient indirect evidence that gut and other microbial agents, for example, *Mycobacterium avium,* are potential triggers for type 1 diabetes [37].

This multifaceted interaction between genetics, immune dysregulation, various infections, and autoimmune diseases such as rheumatoid arthritis (RA) and thyroid disease reveals many possibilities for pathogenic relationships between different species of infectious agents and autoimmunity [20, 38, 39]. These infectious agents and their association with RA and thyroid autoimmunity are shown in Tables 1 and 2.

### 4.1. Mechanisms Responsible for the Induction of Autoimmunity by Infection

Autoimmunity can be induced by infectious agents through the following mechanisms: molecular mimicry, epitope spreading, standard activation, viral persistence, polyclonal activation, dysregulation of immune homeostasis, and autoinflammatory activation of innate immunity [20]. In some cases, even if infections are not directly responsible for the induction of autoimmunities, they can often target the site of autoimmune inflammation and amplify the autoimmune disease [68]. In this case, infections can have one of three effects: first, it can exacerbate ongoing disease, leading to greater severity and duration; second, it can induce a relapse; and, third, it can lead to chronic progressive disease.

#### 4.1.1. Molecular Mimicry

In the most likely mechanism by which infection induces autoimmunity, foreign antigens very often may bear sufficient structural similarity to self-antigens. This is called antigenic mimicry or molecular mimicry. Immune response to microbial antigens could result in activation of T cells that are cross-reactive with self-antigens. This is due to the fact that a single T cell can respond to various peptides with similar charge distribution and overall shape [20, 69]. Examples of bacterial or viral antigens, their cross-reactivity with various tissue antigens, and potential ensuing autoimmune diseases are shown in Table 3.

**Figure 5:**
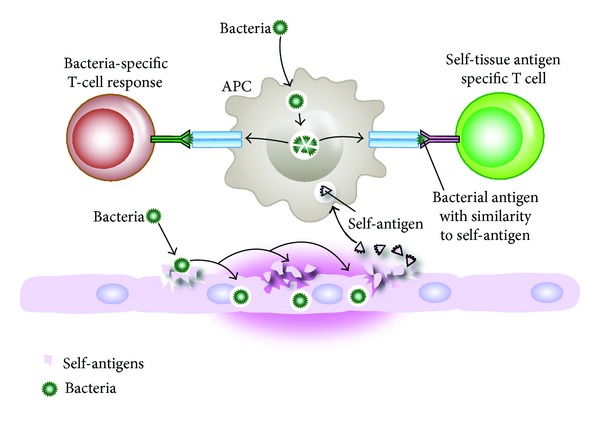
Mechanisms of infection-induced autoimmunity through molecular mimicry. Bacterial induction of self-tissue antigen release and simultaneous presentation of bacterial and self-tissue antigens to T cells; activated T cells can produce antibodies against both bacterial and self-tissue antigens.

Mechanisms of infection-induced autoimmunity through molecular mimicry are shown in Figure 5.

Initiation of immune response to the foreign antigens such as coxsackievirus that share identical amino acid residues with self-proteins such as GAD-65 may generate a cross-reactive antibody response that incorrectly recognizes the self-protein as a foreign antigen. When the self-antigen is a cell surface molecule such as GAD-65, the antibody- and cell-mediated immune response can lead to tissue damage [69].

Given the vast numbers of microbial proteins and their cross-reaction with human proteins, immune response against microbial antigens will not always result in autoimmunity. However, such an initial immune response could result in epitope spreading or exposure of other regions of the same self-protein and production of more antibodies [69]. The criteria for the mechanism of autoimmunity induction were reviewed and summarized by Kivity et al. 2009 [20]. In the classical examples of autoimmunities induced by infections summarized in Table 3, all these criteria are present.

#### 4.1.2. Epitope Spreading

Epitope spreading is a phenomenon in which the immune system expands its response beyond the original epitope recognized by T or B cells to induce the release of non-cross-reactive epitopes that are recognized by the immune system later [70]. Epitope spreading can result from a change in protein structure. One such example is protein citrullination, the changing of an amino acid from arginine to citrulline. This can result not only in immune reaction against the original protein or its citrullinated form, but also against other citrullinated proteins.

**Figure 6:**
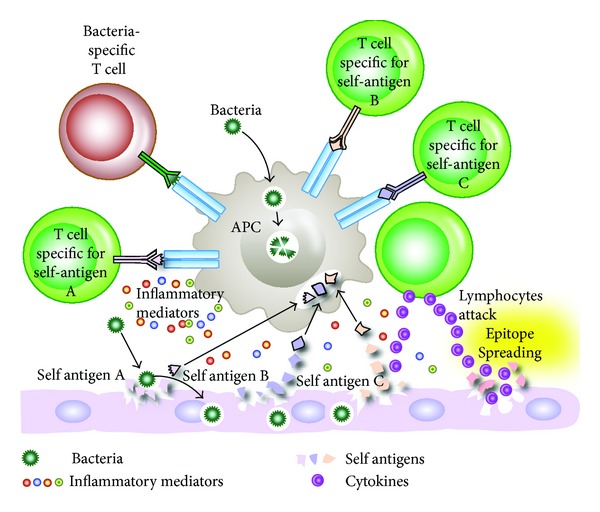
Bacterial infection induces release of tissue antigen and presentation of bacterial and self-tissue antigens resulting in the induction of autoreactive T cells. T cells and inflammatory mediators cause the release of more self-antigens which differ from the original antigens. T-cell responses can then spread to involve T cells specific to other self-antigens. This T-cell response against different epitopes results in antibody production against multiple tissue antigens.

Epitope spreading is demonstrated in rheumatic fever, in which a chronic autoimmune response against streptococcal M protein and heart valve tissue can result in immune response against collagen or laminin. This immune response against collagen or laminin is no longer specific to the bacterial M protein or its cross-reactive tissue protein. In pemphigus, blistering of the mouth precedes blistering of the skin, and blisters in the mouth are associated with the presence of antibodies against desmoglein-3 protein, which is specific to the mouth epithelial cell antigens. It is only later on when T cells attack skin desmoglein-1 that autoantibodies are produced against skin-specific antigens, and skin blistering develops [71]. In a mouse model of encephalomyelitis, Theiler's murine encephalomyelitis virus T-cell response to myelin develops first against dominant myelin proteolipid (PLP) peptide 139–151. As the disease progresses, response to the different and less dominant epitope PLP peptide 178–191 emerges. This mechanism of infection-induced autoimmunity through epitope spreading is shown in Figure 6.

#### 4.1.3. Bystander Activation and Stimulation of Pattern Recognition Receptors

**Figure 7:**
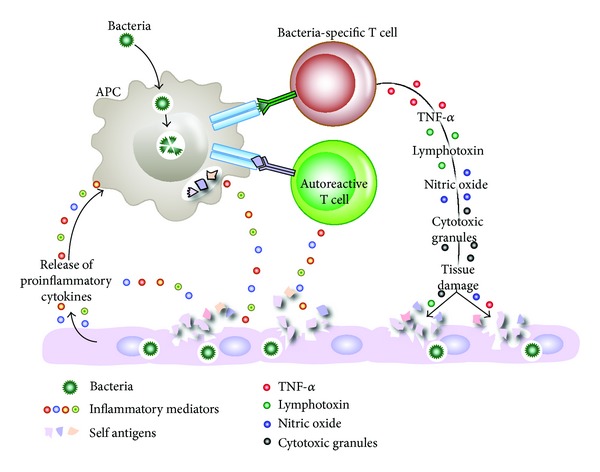
Microbial infection stimulates toll-like receptors (TLRs) and other pattern recognition receptors on antigen-presenting cells (APCs), leading to the production of proinflammatory mediators, which in turn can lead to tissue damage. The release of both tissue antigens and bacterial antigens results in bacterial-specific T cells and autoreactive T cells in the process called bystander activation, which contributes to autoimmunity.

**Figure 8:**
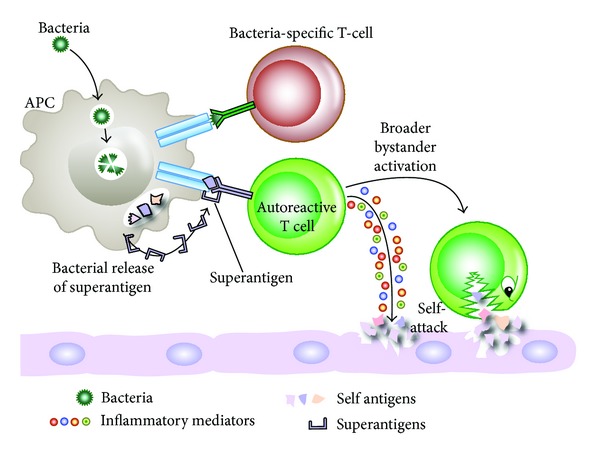
Superantigens and autoimmunity. Infection can lead to the release of superantigens, which can cross-link between MHCII and TCR, causing broader bystander activation, some of which may be specific for self-antigens, leading to attack on self-tissues.

Bystander activation occurs when viral antigens stimulate toll-like receptors and other pattern recognition receptors become activated in the inflammatory environment [72]. This activation of receptors on an antigen-presenting cell (APC) causes the release of proinflammatory cytokines which can induce tissue damage and the release of hidden antigens (Figure 7). The release of tissue antigens can activate autoreactive T cells that initially were not involved in the immune reactivity against the original infection [20]. Additionally, virally infected APCs and the concomitantly released mediators are able to activate autoreactive Th1 or Th17 cells in a bystander manner. Upon recognition of virally infected tissue cells, viral-specific T cells then release cytotoxic granules such as granzymes and cytokines such as TNF-*α*, IL-17, lymphotoxin, and nitric oxide. This inflammatory environment can lead to the bystander killing of uninfected neighboring cells. Microbial superantigens can induce a broader form of bystander activation by cross-linking MHC class II molecules to TCRs on APCs and T-cell activation (Figure 8). T cells that are stimulated in this manner may contain a subset recognizing specific tissue antigen [73]. Examples of superantigens are staphylococcal antigens, mycoplasma antigens, enteric-microbiota LPS, EBV, retrovirus, and many heat shock proteins. Some of these superantigens do not cause autoimmune disease but are involved in the exacerbation of EAE, arthritis, IBD, and other disorders [1].

#### 4.1.4. Persistent Infection and Polyclonal Activation of B Cells

In many autoimmune diseases, such as lupus, RA, type 1 diabetes, and MS, B-cell functions are closely correlated with disease activity. Antibodies produced by B-cell-derived plasma cells contribute significantly to disease pathogenesis [69]. In these and other disorders, prolonged infectivity with a virus such as EBV, viral proteins, or viral genomes can lead to autoimmunity by the constant activation and proliferation of B cells.

**Figure 9:**
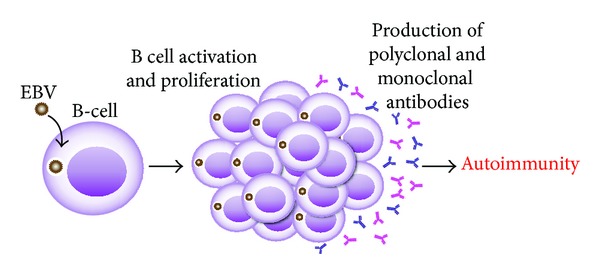
Infections, B cells, and autoimmunity. Prolonged infection with a virus, such as EBV, can lead to constant activation and proliferation of B cells, resulting in the production of monoclonal and polyclonal antibodies as well as immune complexes, causing autoimmune disease.

After a long period of polyclonal B-cell activation, sometimes monospecific clones can emerge, accompanied by very high levels of antibody production and the formation of circulating immune complexes. Finally, this mixture of polyclonal antibodies and immune complexes may cause the autoimmune disease [74], as shown in Figure 9.

## 5. Dietary Components and Autoimmunities

It is undeniable that the diet of the industrialized and urbanized parts of the world today is vastly different from what it was even two or three decades ago, with a whole new range of novel food experiences that come from new food component sources, new breeds of food plants and food animals, genetic modifications, chemical ingredients, flavors, and preservatives. Over recent decades, a significant increase in the incidence of autoimmune diseases such as diabetes and MS in industrialized countries has led to the postulation that diet is a potential environmental risk factor for such disorders. The link between gluten ingestion and gluten sensitive enteropathies is already well established and accepted [3]. High levels of dietary sodium are associated with raised blood pressure and adverse cardiovascular health [75] and have been shown to affect the immune system [76]. Low levels of vitamin D have been linked with MS, systemic lupus erythematosus (SLE), RA, and other autoimmune disorders [3]. Lactose intolerance is no laughing matter for those afflicted with it or other milk-related disorders. The pleasures of a modern diet unfortunately come with caveats and unexpected catches that urgently need investigation.

### 5.1. Sodium Chloride in Diet and Autoimmune Diseases

For the past five decades various studies have been conducted on the comparative sodium intake levels in different countries [75, 76]. Animal experiments, epidemiological studies, and clinical trials have provided convincing evidence for the detrimental effect of sodium intake on blood pressure (BP), coronary heart disease, and stroke, as well as noncardiovascular diseases [77–81]. These comparative studies have shown that generally the simpler and less modernized a society and culture are, the lower the sodium intake is, with a concomitant lessening of the associated disorders. Understandably, the high salt content of the modern Asian diet is known worldwide, particularly the use of soy sauce as a seasoning [75, 82]. Indeed, in comparison to home-made meals, the salt content of fast foods can be many times higher [75]. The concentration of Na^+^ in plasma similar to standard culture medium is about 149 mM. The consumption of high-salt processed foods may increase this concentration to a higher level and result in a change in physiological conditions. It has been theorized that the consumption of processed foods containing high amounts of salt may in part be responsible for the increasing incidence of autoimmune diseases. In a recent study it was demonstrated that an excess uptake of salt can affect the innate immune system, in particular, macrophage function [83]. However, until very recently little was known about increased NaCl intake, its direct effect on the T-helper cell populations, and the connection of all this to autoimmune diseases. Upon stimulation of the T-cell receptor and the cytokine environment, the naïve CD4^+^ T cell can differentiate into functionally distinct effector cell subsets. This differentiation is also driven by key transcriptional regulators such as T-bet for Th1, GATA binding protein-3 for Th2, FOXP3 for Th3, retinoid acid receptor-related orphan receptor gamma t (ROR*γ*t) for Th17, and transcriptional regulator B-cell lymphoma 6 (BCL6) for T-follicular-helper (TFH) cells [84].

**Figure 10:**
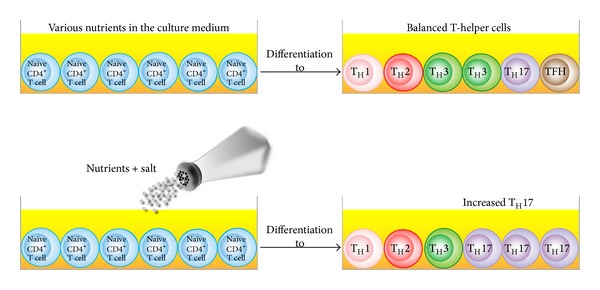
Salt affects the differentiation of naïve CD4^+^ cells. Increased concentrations of salt resulted in the differentiation of naïve CD4^+^ T cells into a greater number of T_H_17 cells.

**Figure 11:**
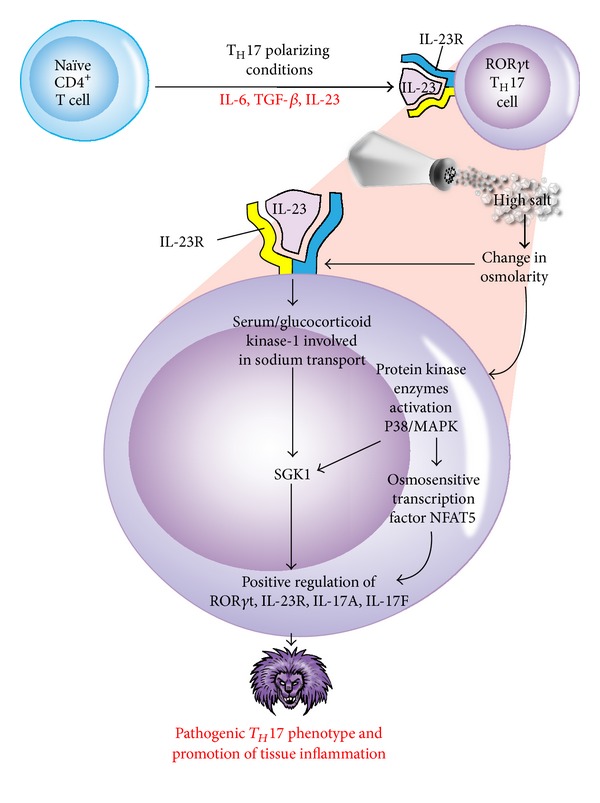
Mechanism by which a high-salt diet enhances the differentiation of naïve CD4^+^ cells to pathogenic T_H_17 cells that may exacerbate experimental autoimmune encephalitis. High salt concentration, change in osmolarity, the influence of IL-23 and IL-23 receptor signaling, and the activation of various enzymes drive the expression of T_H_17-associated cytokines and the formation of pathogenic T_H_17 phenotype.

Among these CD4^+^ T-cell subsets, the IL-23-dependent IL-17-producing CD4^+^ helper T cells play a pivotal role in autoimmune disease [85]. Adding salt to the wound of complex autoimmune diseases, it has been shown that sodium chloride can drive autoimmune disease by the activation or induction of pathogenic Th17 cells [86]. These elegant experiments were conducted in a culture medium containing an additional 10–40 mM concentration of salt, mimicking animals fed a high-salt diet. Increased NaCl concentrations markedly induced the conversion of naïve CD4^+^ T cells to CD4^+^ T cells expressing IL-17A (Figure 10). This effect was dose dependent, and the optimum IL-17A induction was achieved by increasing the concentration of NaCl by 40 mM. Moreover, the authors demonstrated that a high-salt diet could accelerate neuropathology in a mouse model of multiple sclerosis through cellular signaling pathways involving transcription factor NFAT5, the protein kinase enzyme P38, and salt-sensing kinase SGK1. In comparison with the controls, mice on the high-salt diet not only displayed a much higher number of infiltrating CD3^+^ and MAC3^+^ cells but also almost doubled the number of CD4^+^ T cells expressing IL-17A or pathogenic Th17 cells [86, 87]. This effect of the high-salt diet was specific for Th17 conditions, since the high salt levels did not significantly alter cell death, lymphocyte proliferation, or enhancement of Th1 or Th2 differentiations. The mechanism by which a high-salt diet enhances the differentiation of naïve CD4^+^ cells to pathogenic Th17 cells is shown in Figure 11.

**Figure 12:**
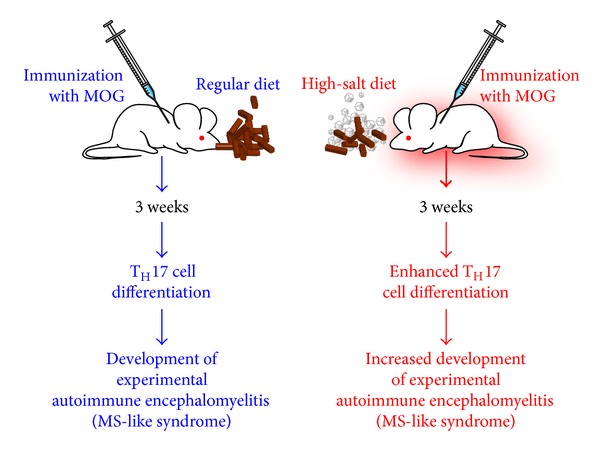
High-salt diet increases risk of autoimmune disease. In two groups of mice, both of which were immunized with MOG to induce EAE, the mice that had been given a high-salt diet (HSD) showed enhanced differentiation of naïve T cells into pathogenic T_H_17 cells and a subsequent increased, more profligate development of EAE.

Extracellular NaCl concentration through the activation of IL-23 receptor and its binding by IL-23 influences the activity of SGK1 and NFAT5 which drives the expression of transcription factor ROR*γ*t, IL-23R, IL-17A, and IL-17F resulting in the phenotype switch from naïve CD4^+^ T cells to pathogenic Th17 cells in MS, psoriasis, and other autoimmune disorders (Figure 12). The data presented in these manuscripts [86, 87] clearly indicate that high intake of sodium potentiates pathogenic Th17 cell generation in *in vitro* and *in vivo* systems in an SGK1-dependent manner and, therefore, has the potential of increasing the risk of promoting autoimmune diseases. Moreover, the elevated *in vivo* Th17 resulting from a high-salt diet raises the important question of whether or not increased salt in westernized diets and in processed foods contributes to an increased generation of pathogenic Th17 cells and towards an unprecedented increase in autoimmune diseases [87].

Thus, as indicated, dietary salt is just one of many dietary components that can influence T-helper cell differentiation and the development of autoimmune disease. The effect of other dietary nutrients, for example, vitamins, and other diverse environmental factors on metabolism and microbiota should also be investigated [88].

When this information is taken together with the strong and consistent evidence that implicates high salt intake with high BP and other cardiovascular disorders, it is alarming to note as laid out in all these studies that most adult populations have daily salt intakes well over the recommended US daily level of 1.5 g/day for middle-aged and older adults [89]. On the face of it a voluntary decrease in salt consumption seems to be an easy policy to implement, but good sense and good health face the formidable opposing forces of flavor, habit, and culture.

### 5.2. The Role of Milk and Wheat Components in Autoimmune Diseases

In relation to dietary proteins it has been well established that different proteins and peptides in milk and wheat are involved in autoimmune diseases [90–93]. Milk contains more than 400 different proteins, most of which have over 150 amino acids (AA). AA that mimic collagen may induce RA, while those that mimic neural cell antigens may induce multiple sclerosis or other neuroimmune disorders.

For example, a study reports that, as a consequence of immunological cross-reactivity or molecular mimicry between the extracellular IV-like domain of the milk protein butyrophilin and myelin oligodendrocyte glycoprotein (MOG), butyrophilin can modulate the encephalitogenic T-cell response to MOG in experimental autoimmune encephalitis [92]. Epidemiological and ecological investigations suggest that early infant nutrition, particularly drinking cow's milk, may induce autoimmunity, leading to type 1 diabetes. This autoimmune reactivity is due to cross-reactivity of cow's milk, particularly its albumin component, with islet cell antigen-1 and beta cell surface protein. These studies suggest that dysregulation of oral tolerance triggers a cellular and humoral immune response against various components of milk proteins, and cross-reaction with B-cell molecules may result in autoimmunity [94–97]. In association with various autoimmune disorders, wheat proteins and, more specifically, gluten, have received significant attention [98–100]. Indeed, it has been demonstrated that a wheat-based diet induces not only Th1-type cytokine bias in the gut but also increased T-cell reactivity to gluten, with a higher frequency of diabetes [99–101]. In addition to diabetes, it has been shown that celiac disease (CD) is associated with various extraintestinal autoimmune disorders that involve the thyroid, joints, heart, skin, pancreas, bone, liver, reproductive organs, and the nervous system [102–112].

Although the exact mechanisms for the induction of these autoimmunities are not definitely known, there is a growing body of evidence indicating that these diseases may result from molecular mimicry between gliadin or transglutaminase and various tissue antigens, including nervous system proteins [33–35, 68]. Interestingly, the celiac peptide VVKVGGSSSLGW shares more than 30% homology with the trangslutaminase peptide 476–487 (RIRVGQSMNMGS) [113]. Therefore, antibodies generated against transglutaminase in the intestine can bind to extraintestinal tissues such as those of the liver, pancreas, lymph nodes, muscle, heart, and brain [114–117]. Very recently, we used both affinity-purified and, monoclonal antibodies against *α*-gliadin 33-mer peptide to examine the cross-reaction between gliadin with different food and tissue antigens [91]. We observed significant immune reactivity when these antibodies were applied to cow's milk, milk chocolate, milk butyrophilin, whey protein, casein, yeast, oats, corn, millet, instant coffee, and rice. With regard to the reaction of *α*-gliadin antibody with various tested tissue antigens, the most significant binding occurred with asialoganglioside, hepatocyte, glutamic acid decarboxylase 65, adrenal 21-hydroxylase, and various neural antigens [92].

These studies collectively indicate that circulating antibodies present in patients with nonceliac gluten sensitivity (NCGS) and CD interact with different food antigens and transglutaminases in various tissues, which may induce the formation of antigen-antibody aggregates that can trigger the activation of the inflammatory cascade.

While most studies about the implications of cross-reactivity with various autoimmunities are limited to milk and wheat, a thorough investigation and understanding of the immunologic cross-reactivity of other food proteins and peptides are essential for advancing our knowledge about the involvement of these dietary components in the development of many autoimmune disorders. Finally, the identification of triggers of autoimmunity can be used in the development of new therapies for autoimmune diseases.

## 6. Using Gluten Sensitivity, Celiac Disease, and Oral Pathogens to Understand Autoimmunities

**Figure 13:**
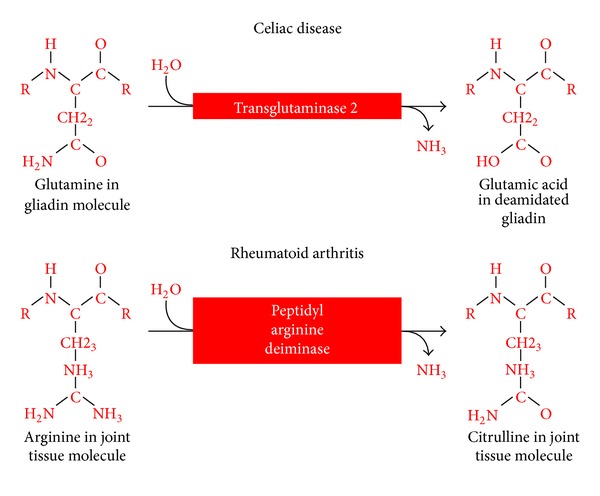
The central role of catalytic enzymes in celiac disease and rheumatoid arthritis. Key enzymes that catalyze the modification of glutamine to glutamic acid or arginine to citrulline as new epitopes have a central role in CD and RA.

There is a lot that clinicians can learn about autoimmune diseases from looking at gluten sensitivity and celiac disease. Some of the features of CD HLA-DQ2/DQ8 association, target organ (villi) T-cell infiltration, and disease-specific autoantibodies produced against modified antigens such as deamidated gliadin and deamidated gliadin-transglutaminase complex [99, 116] are paralleled in chronic joint disorder [94, 118]. These observations suggest that it might be feasible to use CD to identify disease-relevant epitopes in RA and other autoimmune disorders, such as type 1 diabetes and multiple sclerosis [100]. For example, the key enzymes that catalyze the modification of glutamine to glutamic acid or arginine to citrulline as new epitopes have a central role in CD and RA (Figure 13).

**Figure 14:**
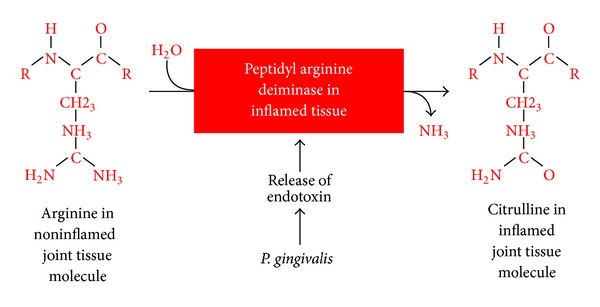
Potentiation by oral pathogens. Oral pathogens such as *P. gingivalis* can potentiate the deamination of arginine or formation of citrullinated proteins and peptides in joint and other tissues.

It is interesting to note that this process of arginine deamination and the formation of citrullinated proteins and peptides in the joint or other tissues could be potentiated by oral pathogens such as *Porphyromonas gingivalis* [40, 119–124] (Figure 14). That is why RA can also cause inflammation in other organs, including the skin, lungs, heart, and peripheral nerves, often with serious consequences.

This is only one observation suggesting that environmental triggers can change self-tissue antigens to become disease-associated T-cell epitope, resulting in antibody production against the citrulline-containing new epitope. Therefore, if CD as an autoimmune disorder is driven by transglutaminase-2 and deamidated gliadin, then we may state that RA is caused by environmental factors, such as *P. gingivalis* or EBV. These environmental factors, by causing the formation of various citrullinated self-epitopes such as collagen type II, fibrin, vimentin, keratin, *α*-enolase, and filaggrin, are involved in the induction of RA [119–125]. Experience with CD has taught scientists that genes, environmental factors, and target tissue antigens are all important issues for consideration in understanding the molecular structure of epitopes recognized by T cells and B cells within the inflamed target organ [100, 124].

### 6.1. Mechanism Involved in the Induction of Autoimmunity by Oral Pathogen

A similar mechanism applies to the autopathogenic correlation of periodontitis induced by *P. gingivalis* and RA [40, 119, 124]. It is possible that both diseases share a common aetiopathogenic background [124]. This mechanism includes the posttranslation modification or citrullination of bacterial proteins and self-antigens simultaneously, generating neoepitope structure. This can result in a breakdown in self-tolerance and antibody production against citrullinated bacterial antigens as well as citrullinated host proteins [125]. One such antigen is *α*-enolase, which features significant homology between human and bacterial *α*-enolase. Therefore, antibodies produced against citrullinated bacterial *α*-enolase will react with human *α*-enolase, and antibodies produced against human citrullinated *α*-enolase will react strongly against bacterial *α*-enolase. For this reason, elevated levels of *α*-enolase antibodies are detected in the synovium of 60% of patients with RA [122]. Indeed, immunological mapping using a library of cyclic citrullinated *α*-enolase peptides led to the identification of a B-cell-dominant epitope comprising amino acids 5–21 of *α*-enolase (KIHAREIFDSRGNPTVE) where arginine-9 and arginine-15 are citrullinated, with an 82% sequence similarity with that of *P. gingivalis* [126, 127]. Immunization with citrullinated human and *P. gingivalis α*-enolase and citrullinated fibrinogen causes similar pathology in humanized DR4 transgenic mice. This mechanism may be the common denominator between autoimmunity and cardiovascular disease. These findings suggest that, by mimicking the molecular structure of host-citrullinated proteins, *P. gingivalis* peptidylarginine deiminase-citrullinated bacterial *α*-enolase could trigger a loss of tolerance to structurally similar host proteins, resulting in expression of anti-citrullinated protein antibodies and the development of RA [128, 129].

These antibodies can be detected up to 10 years before the clinical onset of RA and the production of IgM antibodies against IgG (called rheumatoid factor) in the majority of patients.

**Figure 15:**
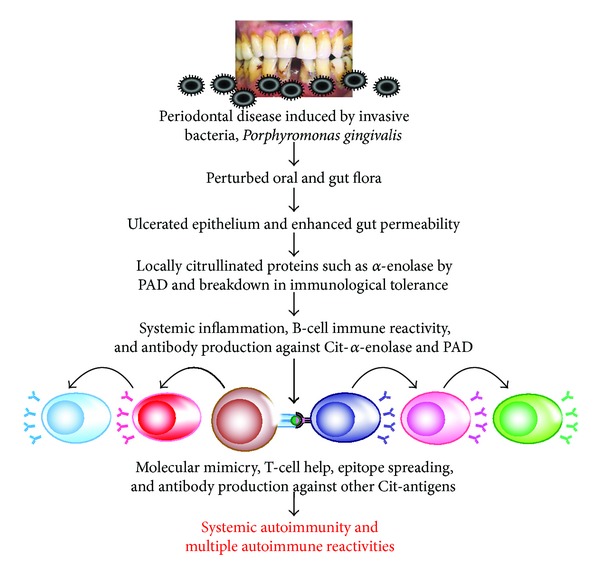
Proposed model for the pathogenesis of multiple autoimmune reactivities by infection. Bacterial generation of autoantigens, local inflammation generating autoantigens by PAD, antibody production against one autoantigen, epitope spreading, antibody production against multiple antigens, systemic inflammation, and multiple autoimmune reactivities.

In the joint, the specificity of anti-citrullinated peptide is enhanced through epitope spreading to other citrullinated autoantigens such as fibrinogen, collagen, filaggrin, and vimentin (see Figure 15).

## 7. Conclusion

Putting all this information together, it appears that there are common mechanisms in the immunopathogenesis of multiple autoimmune reactivities. In genetically susceptible individuals, environmental triggers such as xenobiotics and *P. gingivalis* can, respectively, induce the formation of neoantigens, or be capable of inducing the citrullination of host proteins and converting them to autoantigens. These modified proteins can be recognized by the immune system, triggering antibody production and the inflammatory process involved in the clinical manifestations of autoimmune diseases.

To optimize the chances of therapeutic success it is essential to identify the environmental triggers first and then attempt to remove them from the patient's environment (e.g., toxic chemicals and food associated with autoimmunities). In the case of infections, this also helps to guide the clinical use of various medications which are now often used for prophylaxis. Therefore, careful monitoring for the presence of infections in the patient's blood or tissue will be desirable for monitoring the effects of the drug therapy [40, 119–124].

Manipulation of environment triggers and the host immune system during the clinical and in particular preclinical stages of autoimmune disease will offer significant insight and guide early intervention for many autoimmune disorders that, according to the American Autoimmune Related Disease Association, Inc. (http://www.aarda.org/), affects approximately 10% of the world's population [130], while others put it as high as 20%. Finally, identification of triggers of autoimmunities could be used in the development of new therapies for autoimmune diseases.

## Figures and Tables

**Table 1:** Infectious agents associated with rheumatoid arthritis.

Infection	Reference
*Porphyromonas gingivalis *	Farquharson et al. 2012 [40]
Segmented filamentous bacteria	Wu et al. 2010 [41]
*Yersinia enterocolitica *	Gaston and Lillicrap 2003 [42]
*Salmonella typhi *	McColl et al. 2000 [43]
*Shigella flexneri *	Hannu et al. 2005 [44]
*Proteus mirabilis *	Ebringer and Rashid 2006 [45]
*Campylobacter jejuni *	Pope et al. 2007 [46]
*Klebsiella pneumoniae *	Domínguez-López et al. 2000 [47]
*Clostridium difficile *	Cope et al. 1992 [48]
*Staphylococcus aureus *	Liu et al. 2001 [49]
*Streptococcus pyogenes *	Faé et al. 2006 [50]
*Candida albicans *	Hermann et al. 1991 [51]
*Leptospira pomona *	Sutliff et al. 1953 [52]
*Chlamydia *	Carter et al. 2010 [53]
*Mycoplasma arthritidis *	Cole and Ward 1979 [54]
*Mycobacterium tuberculosis *	Kim et al. 2006 [55]
*Borrelia burgdorferi *	Imai et al. 2013 [56]
*Parvovirus *	Kerr et al. 1995 [57]
Epstein-Barr virus	Pratesi et al. 2006 [58]

**Table 2:** Infectious agents associated with thyroid autoimmunity.

Infection	Reference
*Yersinia enterocolitica *	Bech et al. 1978 [59]
Epstein-Barr virus	Shimon et al. 2003 [60]
*Parvovirus *	Mori et al. 2007 [61]
Hepatitis C	Fernandez-Soto et al. 1998 [62]
Mumps	Parmar et al. 2001 [63]
*Rubella *	Ziring et al. 1977 [64]
*Coxsackievirus *	Brouqui et al. 1991 [65]
HTLV-1	Kawai et al. 1992 [66]
Human herpes virus types 6 and 7	Leite et al. 2008 [67]

**Table 3:** Examples of bacterial and viral antigens that can cross-react with self-antigens with potentially resultant diseases.

Pathogen antigen	Cross-reactive self-antigen	Autoimmune disease
Herpes simplex virus	Corneal antigen	Stromal keratitis
*Campylobacter jejuni *	Ganglioside in peripheral nerve	Guillain-Barré syndrome
Coxsackievirus	Glutamic acid decarboxylase	Type 1 diabetes
Theiler's murine encephalomyelitis virus	Proteolipid protein	Multiple sclerosis
*Yersinia enterocolitica *	Thyrotropin receptor	Thyroid autoimmunity
*Borrelia burgdorferi *	Leukocyte function associated antigen	Lyme arthritis
*Salmonella typhi *and *Yersinia enterocolitica *	HLA-B27	Reactive arthritis
HHV-6, EBV, Rubeolla, influenza virus, and HPV	Myelin basic protein	Multiple sclerosis
Streptococcal M protein	Myosin and other heart valve proteins	Rheumatic fever
*Trypanosoma cruzi *	Cardiac myosis	Chagas heart disease
